# TSH-Secreting Pituitary Macroadenoma in a Girl with Lingual Thyroid

**DOI:** 10.1155/2013/570847

**Published:** 2013-09-12

**Authors:** S. Wacharasindhu, S. Shuangshoti, S. Sunthornyothin

**Affiliations:** ^1^Department of Pediatrics, Faculty of Medicine, Chulalongkorn University, Bangkok 10330, Thailand; ^2^Department of Pathology and Chulalongkorn GenePRO Center, Faculty of Medicine, Chulalongkorn University, Bangkok 10330, Thailand; ^3^Department of Medicine, Faculty of Medicine, Chulalongkorn University, Bangkok 10330, Thailand

## Abstract

Patients with long-standing hypothyroid are, in some cases, reported to develop pituitary gland hyperplasia due to loss of feedback inhibition of thyroxine in hypothalamus—the condition of which typically regresses after thyroxine replacement. Herein, a 15-year-old girl—with long-standing untreated lingual hypothyroid—presents with a pathologically proven TSH pituitary macroadenoma and bilateral large ovarian cysts. Although MR imaging may differentiate between hyperplasia and macroadenoma of the pituitary gland, pathological examination is still a cornerstone to correct diagnosis.

## 1. Introduction

Pituitary gland enlargement due to thyrotroph hyperplasia has been well documented in association with long-standing hypothyroidism. The mechanism can be explained by an overstimulation of thyrotrophs by thyrotropin-releasing hormone (TRH) in classic negative feedback loop. Patients with this condition may be asymptomatic or occasionally present with local neurological pressure effect such as visual field defect or squint. The pituitary gland hyperplasia could be reversible after thyroxine replacement and normalisation of serum TSH. We report, herein, an adolescent girl with undiagnosed long-standing congenital hypothyroidism due to lingual thyroid and pituitary gland enlargement which could not be reversible after thyroxine replacement. Histology confirmed the diagnosis of pituitary gland macroadenoma.

## 2. Case Report

A 15-year-old girl presented with secondary amenorrhea with short stature, abdominal discomfort, and squint. She was born at term with normal birth weight and uneventful postnatal history; nevertheless, growing up, she was considered short compared with girls of the same age. Academically, she was average in class. Her menarche developed when she was 14 years of age; however, secondary amenorrhea had already occurred for 6 months prior to this presentation. On examination, her height and weight were 132 cm and 36 kg, which were on—5 Standard Deviation Score (SDS) and—2 SDS for standard Thai growth chart, respectively. She had breast Tanner III, pubic hair Tanner II. Ophthalmic examination revealed bilateral optic nerve atrophy. Ultrasonography demonstrated bilateral multicystic ovarian masses, 12.8 × 5.6 × 11.3 cm and 12 × 5.9 × 9 cm of the left and right sides, respectively. Free T4 level was 0.08 ng/dL (*N*: 0.8–1.8); TSH >100 mIU/L (*N*: 0.3–4.1); serum prolactin 45.4 ng/mL (*N*: 3–25); FSH 24.5 IU/L; LH <0.1 IU/L; and estradiol 15,469 mmol/L. Ectopic thyroid at sublingual area was detected by technetium thyroid scan. 

**Figure 1:**
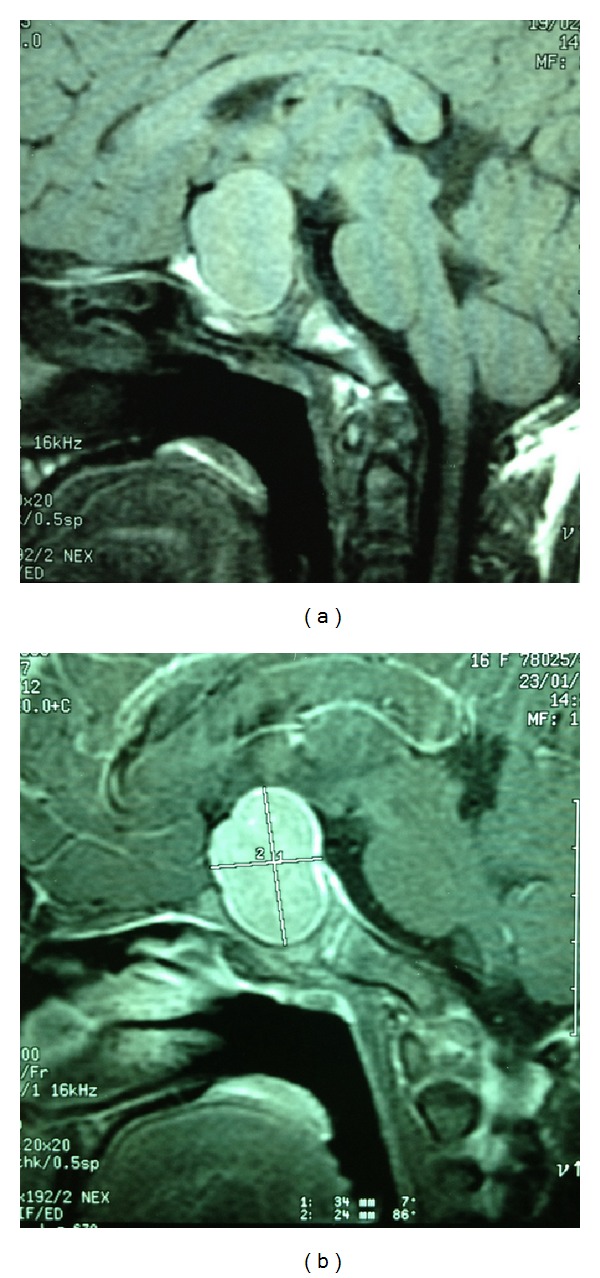
MRI demonstrates a lobulated contour of 3.2 × 4.3 × 3.4 cm mass in sella turcica and suprasellar region (a). After intravenous Gd-DTPA, the mass shows intense enhancement with more intense enhancement in the periphery and central part (b).

Because of the optic nerve atrophy, MRI of the pituitary gland was performed and demonstrated a 3.2 × 4.3 × 3.4 cm heterogeneous enhancing sellar/suprasellar mass (Figure 1). The lesion bulged bilaterally into parasellar areas and cavernous sinuses, more on the left side. Four months after replacing Eltroxin (0.1 mg daily), serum free T4 was 1.74 ng/dL; free T3, 4.83; and TSH, 28.25 uIU/L. Serum prolactin was 35 ng/mL. A repeat MRI showed no significant change of sellar and suprasellar tumors.

**Figure 2:**
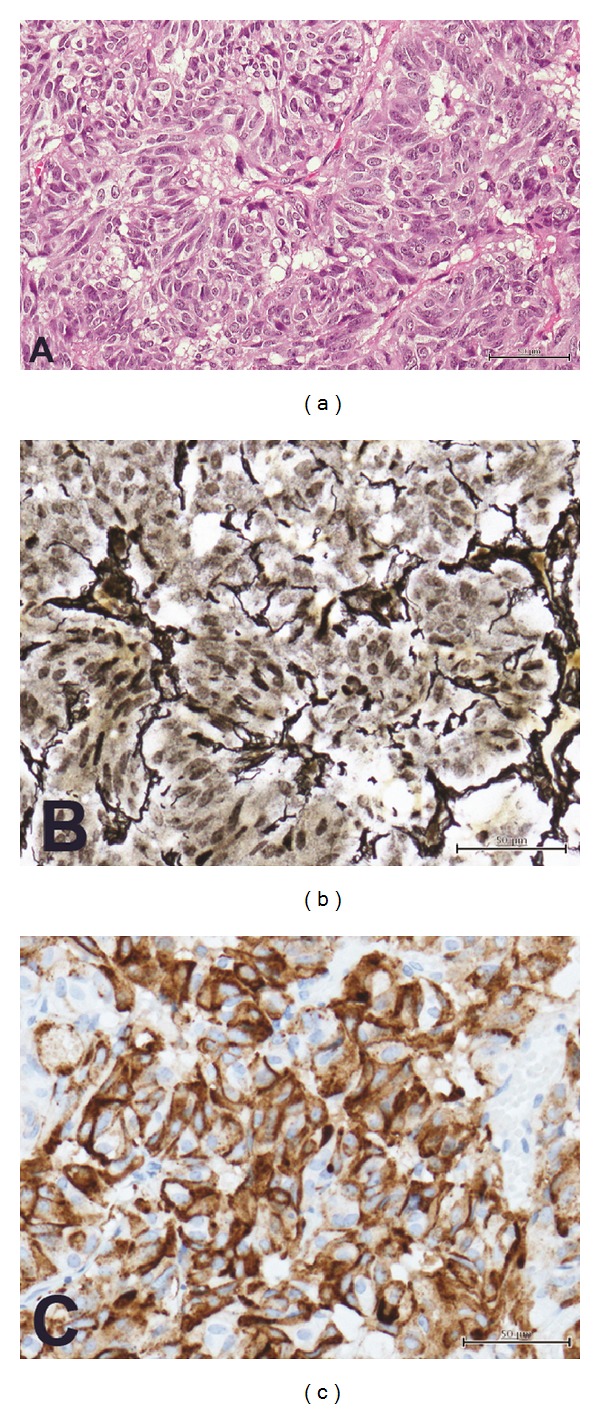
Pathology of TSH pituitary adenoma. Numerous adenoma cells with oval-shaped nuclei are intervened by delicate capillaries (a). Disruption of reticulum framework is depicted (b). Tumor cells are immunoreactive with TSH (c). (A, hematoxylin and eosin; B, reticulin stain; and C, TSH immunostain; each bar = 50 microns).

On a repeat MRI, the mass extended superiorly with pressure effect noted to the optic chiasma and the anterior third ventricle. Due to the compression effect, craniotomy was performed, and the lesion was subtotally removed. Pathologically, monotonous adenoma cells with oval nuclei were found. They were intervened by delicate vasculature (Figure 2(a)). Tumor cells had stippled chromatin pattern, with minute nucleoli and finely granular cytoplasm. Mitotic figures were rarely detected. Disruption of the reticulum architecture, characteristic of pituitary adenoma, was appreciated with reticulin preparation (Figure 2(b)). By immunohistochemical study, tumor cells were diffusely reactive with TSH (Figure 2(c)), while the remaining adenohypophyseal hormonal immunostains were negative. All of the pathological findings were diagnostic of TSH pituitary adenoma. After the operation, Eltroxin and prednisolone were prescribed. Second craniotomy was performed to remove the residual tumor, and radical radiation was administered. After the second surgery, the patient developed hypopituitarism including diabetic insipidus, adrenal insufficiency, and hypogonadotropic hypogonadism. Eltroxin dosage was gradually increased to 0.2 mg daily, and the serum free T4 and TSH were 1.18 ng/dL and 4.6 mIU/L, respectively. Ten months after the second tumor removal, the patient still had no menstruation. Pelvic ultrasonogram showed small uterus with no ovarian follicular development, suggesting hypogonadotropic hypogonadism.

## 3. Discussion 

Pituitary adenoma is a more common cause of intrasellar lesion in adults than in children and can produce various pituitary hormones. TSH-secreting adenoma is, however, rare as it constitutes only 1% of pituitary adenomas [1]. TSH adenomas typically produce hyperthyroidism but may be asymptomatic. Although the patients generally have high T3 and T4 levels with elevated TSH, peripheral thyroid resistance may occur [2, 3]. Pituitary hyperplasia secondary to primary hypothyroid is well described, probably due to loss of thyroxine feedback mechanism and overproduction of TRH. Up to 81% of patients with primary hypothyroidism had increased volume of the sellar on skull X-ray, and sellar volume was positively correlated with the severity of chemical hypothyroidism but not with the duration of symptoms [4, 5]. Desai et al. report ten congenital hypothyroid children, whose mean age at presentation was 11 years and had homogeneous diffuse enlargement of pituitary gland detected by MRI, and all regressed after restoration of hypothyroid status [6]. Adenoma and hyperplasia of the pituitary glands need to be distinguished. In pituitary hyperplasia, the enlarged gland is homogeneously enhanced on MRI study after gadolinium administration. Such enhancing pattern is indistinguishable from the normal gland [7], and it was not seen in our case. In addition, the hyperplastic gland would either disappear or decrease in size after thyroxine replacement [8, 9]. Wolansky et al. demonstrate the disappearance of pituitary gland hyperplasia after 4 months of appropriate thyroxine dose replacement [9]. For the pathological standpoint, enlargement of pituitary lobules with intact reticulum framework features pituitary hyperplasia while, in adenoma, the framework is disrupted as demonstrated in our case [10]. 

Occurrence of TSH macroadenoma in a long-standing untreated hypothyroidism is unusual, and has only been rarely reported, mostly in adult subjects [11, 12]. Myers et al. report a 54-year-old woman with primary hypothyroidism who presented with symptoms of increased intracranial pressure and worsening vision due to thyrotropin-secreting adenoma [13]. In a rare instance, concurrent pituitary hyperplasia and thyrotropin-producing microadenoma are found in association with pituitary resistance to thyroid hormone [2, 3]. 

Multicystic ovarian cyst has been reported in primary hypothyroid and also disappears after thyroid replacement. This association was firstly reported by Wyk and Grumbach [14]. In our patient, hypogonadotropic hypogonadism was detected, which may be due to compression effect of macroadenoma or to surgical damage around the pituitary area. TSH adenomas may need medical treatment such as somatostatin, bromocriptine, or cabergoline. Surgery is indicated if the optic nerve is compressed. Recurrence can be managed with reoperation, radiation, and/or medication.

In conclusion, the study reports an adolescent girl with long-standing, undetected lingual hypothyroidism and TSH-secreting pituitary macroadenoma. It demonstrates that TSH-secreting pituitary adenoma should be considered in patients with persistent high TSH levels in primary hypothyroidism after thyroxine replacement.
